# Flavonol 7-*O*-Glucoside Herbacitrin Inhibits HIV-1 Replication through Simultaneous Integrase and Reverse Transcriptase Inhibition

**DOI:** 10.1155/2019/1064793

**Published:** 2019-02-03

**Authors:** Éva Áy, Attila Hunyadi, Mária Mezei, János Minárovits, Judit Hohmann

**Affiliations:** ^1^National Public Health Institute, Department of Retroviruses, National Reference Laboratory of HIV, 1097 Budapest, Hungary; ^2^Institute of Pharmacognosy, Interdisciplinary Excellence Centre, University of Szeged, 6720 Szeged, Hungary; ^3^Interdisciplinary Centre of Natural Products, University of Szeged, 6720 Szeged, Hungary; ^4^Department of Oral Biology and Experimental Dental Research, Faculty of Dentistry, University of Szeged, 6720 Szeged, Hungary

## Abstract

Here we report the evaluation of the antiretroviral effect of two flavonoid 7-*O*-glucosides, herbacitrin (**1**) and gossypitrin (**2**), together with quercetin (**3**), a well-studied flavonol. Antiviral activity of the flavonoids was assessed by analyzing HIV-1 p24 core protein levels in the supernatants of HIV-1 infected MT-4 and MT-2 cell cultures. The compounds showed mild to weak cytotoxic activities on the host cells; herbacitrin was the strongest in this regard (CC_50_=27.8 and 63.64 *μ*M on MT-4 and MT-2 cells, respectively). In nontoxic concentrations, herbacitrin and quercetin reduced HIV-1 replication, whereas gossypitrin was ineffective. Herbacitrin was found to inhibit reverse transcriptase at 21.5 *μ*M, while it was a more potent integrase inhibitor already active at 2.15 *μ*M. Therefore, our observations suggest that herbacitrin exerts antiretroviral activity through simultaneously acting on these two targets of HIV-1 and that integrase inhibition might play a major role in this activity.

## 1. Introduction

Human immunodeficiency virus type 1 (HIV-1) is the causative agent of acquired immune deficiency syndrome (AIDS). There are approximately 37 million people currently infected with HIV worldwide. In the last decades, more than two-dozen new drugs were approved for clinical use against HIV. Combination antiretroviral therapy (cART) uses different classes of drugs that act in concert to curb HIV replication. The major classes are protease inhibitors (PIs), nucleoside and nonnucleoside reverse transcriptase inhibitors (NRTIs/NNRTIs), entry inhibitors (CCR5 coreceptor antagonists, fusion inhibitors, and postattachment inhibitors), and integrase inhibitors (INIs) [1–3]. In 1996, the combination of antiretroviral drugs was introduced as a highly active antiretroviral therapy (HAART), which transformed HIV/AIDS from a life-threatening condition to a manageable disease [4]. However, the need for lifelong treatment, the severe side effects, and the presently unknown long-term effects of this therapy still represent serious problems. In addition to this, drug resistance can also emerge due to the low genetic barrier allowing related mutations of the virus [5]. Consequently, there is still a need for the development of novel drugs for efficient antiretroviral therapy.

It should also be noted that even though only limited evidence supports this practice, complementary and alternative medicine is used worldwide to treat HIV [6–8]. Traditional herbal medicine is particularly popular in this regard in the African continent, where it frequently appears as the sole therapeutic approach in rural communities [9]. While ineffective, non-evidence-based treatment of HIV represents a serious healthcare problem and a risk to all the surrounding community, plant secondary metabolites undoubtedly deserve much attention when searching for new therapeutic approaches.

Natural products offer a great pool of promising candidates for finding new lead compounds against HIV, and flavonoids appear to be particularly promising in this regard: they can inhibit a wide variety of viral and cellular enzymes participating in the life cycle of HIV, such as reverse transcriptase (RT), integrase (IN), viral protease (PR), and casein kinase II, a cAMP-, cGMP-, and Ca^2+^/phospholipid-independent serine/threonine protein kinase [10, 11].

Previous studies showed that different types of flavonoids, especially certain flavonols, flavones, isoflavones, catechin derivatives, and chalcones, can act as multitarget agents through simultaneously inhibiting crucial enzymes of HIV-1 (RT, IN, and PR) and also interfering with different steps of the virus' life cycle [11]. In this regard, the most studied flavonoid is quercetin that was reported to exert significant anti-HIV activity by inhibiting HIV replication and to reduce virus infectivity in normal peripheral blood mononuclear cells (PBMC) [12]. Inhibition of syncytium formation and protection of HIV-1 induced cytopathic effects by quercetin in C8166 cells has also been reported with EC_50_ values of 42.55 and 23.2 *μ*g/ml, respectively. Quercetin showed antiviral effect with IC_50_ values between 29.76-88.98 *μ*M when tested on TZM-bl cell plus HIV-1 BaL and H9, and PBMC plus HIV-1 MN [13].

In 1994, Fesen et al. reported the screening and SAR study of 48 flavonoids, including hydroxyl- and methoxy-substituted flavones and flavonols, and some glycosides, together with kinetic studies on the relative inhibition of the processing and strand transfer steps [14]. Several further, related studies were performed concerning the IN inhibitory activity of flavonoids as well, and considerable efforts were put into the development of predictive* in silico* screening tools [15]. In such a study, quercetagetin (6-hydroxyquercetin) was identified as a strong inhibitor of viral cleavage and integration [16].

In 2002, three IN inhibitor flavonoids were isolated from the marine organism* Thalassia testudinum,* a Caribbean Sea grass, namely thalassiolins A-C expressing a unique flavone 7-*β*-d-glucopyranosyl-2′′-sulfate structure. Thalassiolin A displayed* in vitro* antiretroviral activity against the strand transfer reaction with a submicromolar inhibitory concentration, and it could inhibit HIV infection of MT-2 cells with an IC_50_ value of ca. 30 *μ*M while exerting no cytotoxicity at concentrations as high as 800 *μ*M [17].

**Figure 1:**
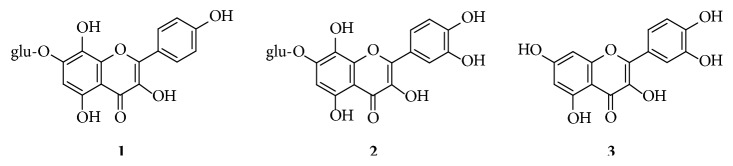
Structures of herbacitrin (**1**), gossypitrin (**2**), and quercetin (**3**).

The above findings suggest that quercetin analogs, and especially 7-*O*-glycosylated compounds with an additional hydroxyl group at their ring A, are worthy of studying as antiretroviral agents. In pursuing this notion, we selected two such compounds, herbacitrin (**1**) and gossypitrin (**2**) (Figure 1), constituents of many Asian medicinal plants, for investigating their* in vitro* cytotoxicity, anti-HIV-1 activity, and reverse transcriptase and integrase inhibitory activity. The data were compared with those of quercetin, which therefore served as a well-established positive control in our study presented hereinafter.

## 2. Results and Discussion

Flavonoid 7-*O*-glucosides, herbacitrin (**1**) and gossypitrin (**2**) (Figure 1) were investigated for their antiretroviral activity in comparison with quercetin, a well-studied abundant flavonol. Herbacitrin (**1**) and gossypitrin (**2**) have been first isolated from cotton flowers (*Gossypium herbaceum*), [18] and later both compounds were detected in different* Equisetum* species [19, 20]. Gossypitrin was also identified in yellow petals of* Papaver nudicaule*, [21] and flowers of* Talipariti elatum *[22].

*Drosera peltata* (shield sundew), a species distributed in India and Southeast Asia, was found to contain both herbacitrin and gossypitrin; this plant is used as an antitussive in the phytotherapy [23]. The antibacterial and antifungal activities of gossypitrin were recently demonstrated against a series of microorganisms, [24] but, to the best of our knowledge, no previous studies are available concerning the antiviral effect of herbacitrin or gossypitrin.

Before the bioassays, herbacitrin (**1**) and gossypitrin (**2**) were subjected to NMR measurements with the aim of assessing the purity of the compounds; this was found to be higher than 90%. Moreover, as a result of our NMR studies, previously unpublished ^1^H and ^13^C chemical shift assignments were also achieved in CD_3_OD, as listed in the Materials and Methods section.

To ascertain nontoxic working concentrations of the flavonoid derivatives, the compounds' cytotoxicity was determined on MT-4 and MT-2 cell lines by MTT assay. Herbacitrin, gossypitrin, and quercetin decreased the cell viability in a dose-dependent manner. The 50% cytotoxic concentrations (CC_50_) of herbacitrin, gossypitrin, and quercetin on the MT-4 and MT-2 cells are presented in Table 1.

**Figure 2:**
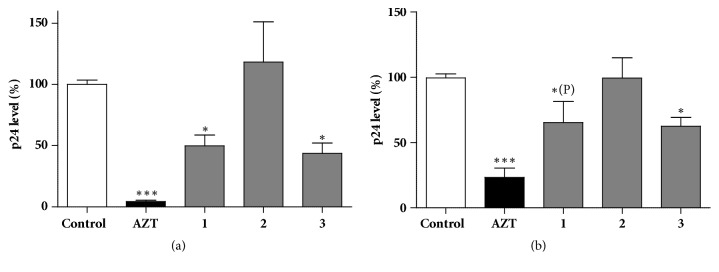
Effect of noncytotoxic concentrations of herbacitrin (**1**), gossypitrin (**2**), and quercetin (**3**) on HIV-1 replication in MT-4 (a) or MT-2 (b) cells cultivated* in vitro*. Compounds** 1**-**3** were applied at 2.1 *μ*M, AZT: azidothymidine (nucleoside reverse transcriptase inhibitor; positive control at 0.64 *μ*M). Means are given in percentage of the virus control; the error bars show standard error of mean (SEM); statistically significant differences were evaluated as compared to the negative control: *∗* and *∗∗∗*: p<0.05 and 0.001, respectively, by means of one-way ANOVA followed by Bonferroni's post hoc test, *∗*(P): p<0.05 by means of a planned comparison, involving compound** 1** and the positive and negative controls only, by one-way ANOVA and Bonferroni's post hoc test.

The antiviral activity of flavonoid derivatives was evaluated by analyzing HIV-1 p24 core protein levels in the supernatants of HIV-1 infected MT-4 and MT-2 cell cultures after 5 days of incubation. HIV-1 infected, untreated cells and HIV-1 infected cells treated with azidothymidine (AZT, a potent nucleoside reverse transcriptase inhibitor) were used as controls. In nontoxic concentrations, herbacitrin and quercetin reduced HIV-1 replication, whereas gossypitrin was ineffective (Figure 2).

**Figure 3:**
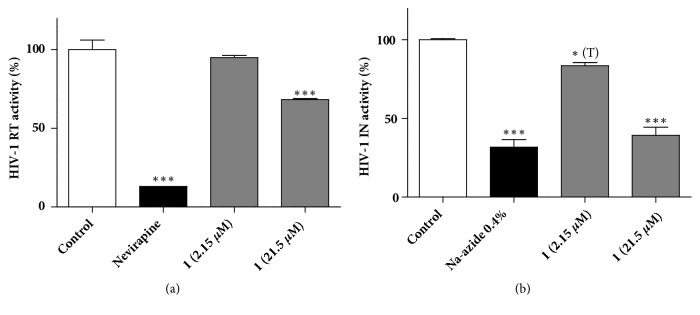
Effect of noncytotoxic concentrations of herbacitrin (**1**) on HIV-1 reverse transcriptase (a) or integrase (b) activity. Positive control: Nevirapine (nonnucleoside RT inhibitor, 18.8 *μ*M) or sodium azide. Means are given in percentage of the negative control, the error bars show standard error of mean (SEM); *∗∗∗*: p<0.001 by means of one-way ANOVA followed by Bonferroni's post hoc test, *∗*(T): p<0.05 by means of a planned comparison by unpaired T-test, as compared to the negative control.

To determine the potential target of herbacitrin within the HIV-1 replication cycle, we tested its effect on the activity of recombinant HIV-1 reverse transcriptase (RT) and integrase (IN). We observed that herbacitrin, applied at a relatively high, 21.5 *μ*M concentration, significantly inhibited the HIV-1 reverse transcriptase (Figure 3(a)). In contrast, the activity of integrase was inhibited already at a lower, 2.15 *μ*M concentration of herbacitrin (Figure 3(b)). These results suggest that herbacitrin may interfere with the replication cycle of HIV at multiple stages.

While the three compounds tested in this study allow only a limited evaluation of structure-activity relationships, from a comparison of the activity of compounds** 1** and** 2,** it appears to be clear that a catechol moiety in the flavonoid B-ring (as in compound** 2**) is unfavorable concerning the anti-HIV activity of flavonol 7-*O*-glycosides. This, however, does not apply to the aglycone quercetin that contains such a catechol B-ring and that was found similarly potent as herbacitrin against HIV replication in our experimental setup. Previously, much higher, one or even nearly two orders of magnitude higher concentrations of quercetin were reported as necessary for significant activity [12, 13]. Concerning the role of the sugar part, the presence of a 3-glycoside moiety, as in myricetin, was previously suggested to assist the internalization of a flavonoid into the cell, hence increasing its ability to interfere with HIV [25]. As of now, however, no related studies are available on the role of a 7-glycoside moiety.

## 3. Conclusion

To the best of our knowledge, this is the first report on the anti-HIV activity of the flavonoid 7-*O*-glycoside herbacitrin. This compound may inhibit HIV-1 replication predominantly by targeting the HIV-1 integrase enzyme. Herbacitrin is a major flavonoid of the flowers found in* Gossypium hirsutum*, a widely used traditional herbal medicine. While we could not find track of HIV treatment-related traditional use of cotton flowers in the scientific literature, our results might warrant investigating a possible positive effect on HIV-infected patients treated with such preparations for other indications. At the same time, the anti-HIV activity of herbacitrin strongly justifies further studies on 7-*O*-glycosylated, noncatechol flavonols against HIV-1, as well as on traditional herbal preparations containing significant amounts of such constituents.

## 4. Materials and Methods

### 4.1. General

NMR spectra were recorded in MeOH-*d*_4_ on a Bruker Avance DRX 500 spectrometer at 500 MHz (^1^H) or 125 MHz (^13^C); the signals of the deuterated solvent were taken as reference. Two-dimensional (2D) experiments (^1^H-^1^H COSY, HSQC and HMBC) were set up, performed, and processed with the standard Bruker protocol. Herbacitrin and gossypitrin were purchased from Atomax Chemicals Co., Ltd. (Shenzhen, Guangdong, China) purity >90%, and quercetin from Sigma-Aldrich (Saint Louis, Missouri, USA) purity >98%.

### 4.2. Herbacitrin (**1**)

^1^H-NMR (500 MHz, CD_3_OD): *δ*_H_ 6.67 (1H, s, H-6), 8.21 (2H, d,* J*=8.7 Hz, H-2′, 6′), 6.93 (2H, d,* J*=8.8 Hz, H-3′, 5′), 4.97 (1H, d,* J*=7.6 Hz, H-1′′), 3.55 (1H, m, H-2′′), 3.50 (1H, m, H-3′′), 3.44 (1H, m, H-4′′), 3.48 (1H, m, H-5′′), 3.92 (1H, dd,* J*=12.1, 1.5 Hz, H-6′′a), 3.75 (1H, dd,* J*=12.2, 4.9 Hz, H-6′′b). ^13^C-NMR (125 MHz, CD_3_OD): *δ*_C_ 149.0 (C-2), 137.3 (C-3), 177.8 (C-4), 153.6 (C-5), 99.9 (C-6), 151.8 (C-7), 129.0 (C-8), 145.7 (C-9), 106.6 (C-10), 123.9 (C-1′), 131.1 (C-2′, 6′), 116.4 (C-3′, 5′), 160.8 (C-4′), 103.5 (C-1′′), 74.9 (C-2′′), 77.7 (C-3′′), 71.3 (C-4′′), 78.5 (C-5′′), 62.4 (C-6′′).

### 4.3. Gossypitrin (**2**)

^1^H-NMR (500 MHz, CD_3_OD): *δ*_H_ 6.67 (1H, s, H-6), 7.85 (1H, d,* J*=2.1 Hz, H-2′), 6.90 (1H, d,* J*=8.6 Hz, H-5′), 7.77 (1H, dd,* J*=8.6, 2.1 Hz, H-6′), 4.96 (1H, d,* J*=7.6 Hz, H-1′′), 3.57 (1H, t,* J*=8.8 Hz, H-2′′), 3.52 (1H, m, H-3′′), 3.42 (1H, m, H-4′′), 3.48 (1H, m, H-5′′), 3.91 (1H, dd,* J*=12.1, 1.9 Hz, H-6′′a), 3.75 (1H, dd,* J*=12.1, 4.9 Hz, H-6′′b). ^13^C-NMR (125 MHz, CD_3_OD): *δ*_C_ 148.9 (C-2), 137.4 (C-3), 177.8 (C-4), 153.5 (C-5), 99.9 (C-6), 151.7 (C-7), 129.0 (C-8), 145.7 (C-9), 106.6 (C-10), 124.3 (C-1′), 116.4 (C-2′), 146.3 (C-3′), 149.0 (C-4′), 116.3 (C-5′), 122.3 (C-6′), 103.5 (C-1′′), 74.9 (C-2′′), 77.7 (C-3′′), 71.3 (C-4′′), 78.5 (C-5′′), 62.4 (C-6′′).

### 4.4. Cells and Virus

The permanent human T-cell lines MT-4 and MT-2 were maintained at 37°C in a humidified atmosphere containing 5% CO_2_ in RPMI 1640 (Sigma-Aldrich) medium supplemented with 10% heat-inactivated fetal bovine serum (Sigma-Aldrich), 100 IU/ml penicillin and 100 *μ*g/ml streptomycin (Sigma-Aldrich). HIV-1 (HTLV-IIIB) was obtained from the culture supernatant of MT-4/HTLV-IIIB cells. The 50% HIV-1 tissue culture infectious dose (TCID_50_) on MT-4 cells was determined by virus yield assay [26]. The titer of the virus stock was 2.32*∗*10^5^ TCID_50_/ml.

### 4.5. Cytotoxicity Assay

To determine the* in vitro* cytotoxic effect of the compounds, viability of the treated and untreated cells was measured by a colorimetric assay as described earlier [27]. Briefly, MT-4 and MT-2 cells were seeded into a 96-well plate at a density of 15,000 cells/well in the presence of different concentrations of the compounds dissolved in dimethyl sulfoxide (DMSO). The final concentration of DMSO used in the experiments did not affect the cell viability. After 4 days of incubation, cell cultures were analyzed using MTT cell viability assay (Sigma-Aldrich) to monitor the reduction of 3-(4,5-dimethylthiazol-2-yl)-2,5-diphenyl-tetrazolium bromide (MTT) to a blue formazan product by metabolically active cells. To initiate the cell viability assay, 20 *μ*l MTT (5 mg/mL dissolved in PBS) was added to each well. After 4 h incubation cell supernatant was removed and 100 *μ*l DMSO per well was added. The absorbance was measured at 550 nm on a microplate reader after mixing the contents thoroughly. The cytotoxicity tests were implemented in two biological replicates. The CC_50_ (50% cytotoxic concentration) values were determined by nonlinear regression using the variable slope log (inhibitor) vs. normalized response model of GraphPad Prism 5 (GraphPad Software, San Diego, CA, USA).

### 4.6. Cell-Based Antiviral Assay

MT-4 and MT-2 cells at a density of 15,000 cells/well were incubated in 96-well plates in the presence of compounds at 37°C in 5% CO_2_ for 5 days. Simultaneously, cells were exposed to HIV-1 (2,32*∗*10^2^ TCID_50_/ml). Untreated and infected or AZT (3′-azido-3′-deoxythymidine)-treated cells were used as controls. After the incubation period, diluted culture supernatants were analyzed for HIV production by determining the amount of viral core protein using a p24 enzyme-linked immunosorbent assay (ELISA) kit (Fujirebio) according to manufacturer's instructions. The results were expressed relative to the control of untreated HIV-1 infected cells. The experiment was performed in four biological replicates. Statistical analysis was performed by one-way ANOVA followed by Bonferroni's post hoc test.

### 4.7. HIV RT and IN Inhibition Assays

Inhibitory effects of compounds on the HIV-1 reverse transcriptase and integrase activity were measured by a colorimetric RT kit (Roche Diagnostics) and IN assay kit (Express Biotech International) according to the instructions of the manufacturer. Reverse transcriptase assay measures the amount of labeled nucleotides incorporated during the transcription process of RNA. Nevirapine, a nonnucleoside RT inhibitor, was used as a positive control in the RT reaction. HIV-1 integrase assay measures the integrase activity after 3′-end processing of the HIV-1 LTR donor substrate DNA and catalyzing the strand-transfer recombination reaction to integrate the donor substrate DNA into the target substrate DNA. Sodium azide was applied as a positive control compound in the experiments measuring the integrase activity. The RT and IN inhibition assays were performed in two biological replicates. Statistical analysis was performed by one-way ANOVA followed by Bonferroni's post hoc test, and a planned comparison by unpaired T-test was also performed (see Figure 3(b)).

## Figures and Tables

**Table 1:** Cytotoxicity of herbacitrin, gossypitrin, and quercetin on MT-4 and MT-2 cells. CC_50_: concentration that causes 50% cytotoxicity, C.I.: 95% confidence interval for the CC_50_ values obtained from the nonlinear curve fitting, n=4.

Compound	CC_50_ (*μ*M) [95% C.I.]	
	MT-4	MT-2
Herbacitrin (**1**)	27.8 [26.79-28.79]	63.64 [57.50-70.41]
Gossypitrin (**2**)	101.0 [90.83-104.98]	112.56 [100.29-126.35]
Quercetin (**3**)	107.5 [97.22-118.97]	157.38 [136.75-181.09]

## Data Availability

Underlying data related to this submission are available from the authors.
